# A dynamic interplay between phytohormones is required for fruit development, maturation, and ripening

**DOI:** 10.3389/fpls.2013.00079

**Published:** 2013-04-17

**Authors:** Peter McAtee, Siti Karim, Robert Schaffer, Karine David

**Affiliations:** ^1^School of Biological Sciences, The University of AucklandAuckland, New Zealand; ^2^The New Zealand Institute for Plant and Food Research LimitedAuckland, New Zealand

**Keywords:** fruit development, ripening, hormonal regulation

## Abstract

Plant species that bear fruit often utilize expansion of an ovary (carpel) or accessory tissue as a vehicle for seed dispersal. While the seed(s) develop, the tissue(s) of the fruit follow a common progression of cell division and cell expansion, promoting growth of the fruit. Once the seed is fully developed, the fruit matures and the surrounding tissue either dries or ripens promoting the dissemination of the seed. As with many developmental processes in plants, plant hormones play an important role in the synchronization of signals between the developing seed and its surrounding fruit tissue(s), regulating each phase of fruit development. Following pollination, fruit set is achieved through a de-repression of growth and an activation of cell division *via* the action of auxin and/or cytokinin and/or gibberellin. Following fruit set, growth of the fruit is facilitated through a relatively poorly studied period of cell expansion and endoreduplication that is likely regulated by similar hormones as in fruit set. Once the seeds reach maturity, fruit become ready to undergo ripening and during this period there is a major switch in relative hormone levels of the fruit, involving an overall decrease in auxin, gibberellin, and cytokinin and a simultaneous increase in abscisic acid and ethylene. While the role of hormones in fruit set and ripening is well documented, the knowledge of the roles of other hormones during growth, maturation, and some individual ripening components is sketchy.

## BACKGROUND

**FIGURE 1:**
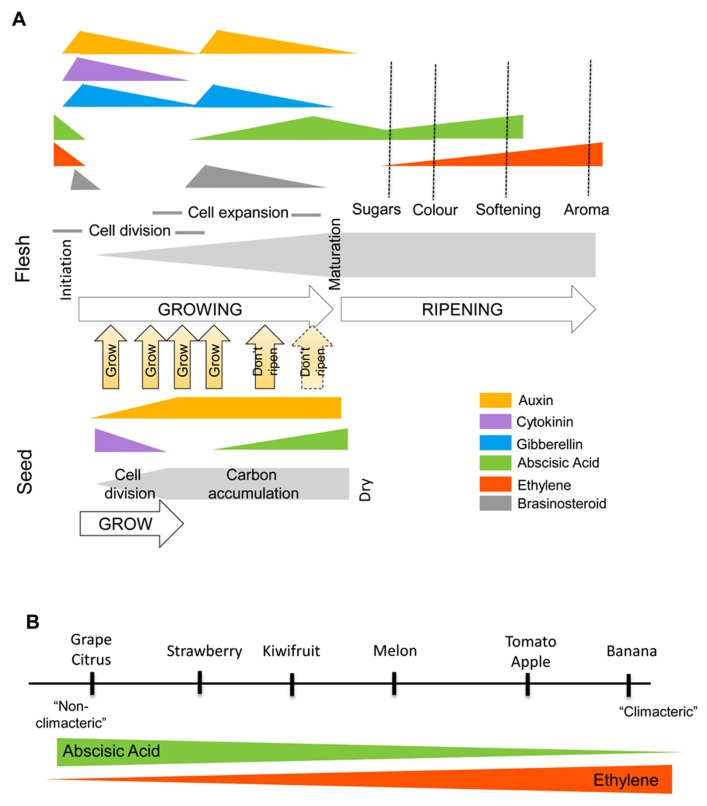
**(A)** Hormonal changes that occur in a generic fruit during development and ripening. Differential hormone concentrations occur in the seed and the surrounding tissue with the developing seed influencing its environment. Multiple studies have shown that increases in auxin, cytokinin, gibberellin, and brassinosteroid at fruit set, and an involvement of auxin, gibberellin, and brassinosteroid at fruit growth. For fruit maturation there is an inhibition of auxin transport from the seed and increase in ABA. This triggers the ripening/senescence program which leads to an increase in ABA and/or ethylene biosynthesis and response in the surrounding tissue. **(B)** The spectrum of ripening dependencies to ABA and ethylene. All fruit appear to respond to ABA and ethylene. In historically considered “climacteric fruit,” ABA indirectly regulates ripening through ethylene. In “non-climacteric” fruit, the ABA has a more dominant role but the fruit still have ethylene-dependant ripening characters.

The fruiting body of flowering (angiosperm) plants has evolved to best aid seed protection and dispersal. A diverse range of fruit types within angiosperm species exists and these variations are exemplified between fleshy fruits, that have evolved with an enlargement of the tissue surrounding the seed to create attractive flesh for seed dispersing animals, and “dry” fruit, that split open (dehisce) to release the seed *via* abiotic dispersal mechanisms. Evolutionary studies have revealed that plant species producing fleshy fruit have evolved from ancestral dry fruit producing species, suggesting common mechanisms between dry and fleshy fruit (Knapp, 2002). Pulling upon the literature from across different species, we have revealed common trends in the hormonal regulation of the different stages of fruit development (**Figure 1**). In all cases, dry or fleshy fruit undergo a progression of specific steps including: fruit set, fruit growth, maturation, and ripening/senescence. The crosstalk between hormones that occurs during most of these steps is scarce, nevertheless with the advent of genomic and high throughput technologies there has been significant progress in characterizing hormones and the expression of associated downstream genes in both model and non-model organisms. In this review, we aim to give an overview of the way plant hormones interact to control these different developmental steps and the switch(es) between them as well as highlight areas that require further research to understand these complex processes.

## FRUIT SET

Fruit set is the first step in fruit development; it is established during and soon after fertilization. Seed bearing plants have a unique double fertilization event with two pollen nuclei fertilizing the embryo and the endosperm (Dumas et al., 1998; Raghavan, 2003; Hamamura et al., 2012). The role of hormones during embryo development and seed maturation has been well reviewed (for example: Gutierrez et al., 2007; Sun et al., 2010). The fertilization event leads to the development of the seed that de-represses cell division and fruit growth in a synchronized manner (review: Fuentes and Vivian-Smith, 2009). Fruit set has traditionally been attributed to the action of three hormones, auxin, and/or gibberellin, and/or cytokinin (Mariotti et al., 2011). Application of these hormones alone can trigger fruit development to a certain extent and, in many plant species, application in combination will induce normal fruit growth even in the absence of fertilization (parthenocarpy; Nitsch, 1952; Crane, 1964; Gillaspy et al., 1993; Vivian-Smith and Koltunow, 1999), indicating that an interplay between these hormones is necessary for fruit set and fruit growth. In many species, auxin and cytokinin levels in the seed increase during seed development until maturity (Nitsch, 1950; Blumenfeld and Gazit, 1970; Varga and Bruinsma, 1976; Yang et al., 2002; Devoghalaere et al., 2012) and in pea, removal of the seed leads to reduced gibberellin biosynthesis in the pericarp (García-Martínez and Carbonell, 1980; Ozga et al., 1992). These observations led to the “seed control” hypothesis where the seeds communicate through hormones to the surrounding tissue(s) to promote fruit growth through firstly cell division and later on cell expansion (Ozga et al., 2002).

At the molecular level, the main advances have been on how gibberellin and auxin pathways interact to promote fruit set in both dry fruit, such as *Arabidopsis thaliana* (*Arabidopsis*), and fleshy fruit, such as tomato (de Jong et al., 2009a; Carrera et al., 2012; Ruan et al., 2012). Early studies showed that elevated levels of gibberellins and auxin are present in fruits from plants that exhibit parthenocarpy (Talon et al., 1990) and auxin levels increase during seed development while gibberellin levels increase in the ovaries following fertilization (Olimpieri et al., 2007; Hu et al., 2008). In *Arabidopsis*, fruit development induced by auxin occurs solely through activation of gibberellin signaling and the current, simplified model, of auxin and gibberellin action is the following: auxin, synthesized in the ovules on fertilization is transported to the pericarp where it induces gibberellin biosynthesis (Zhao, 2010). In turn, the newly synthesized gibberellin will lead to the release of growth repression (Fuentes et al., 2012). There are additional layers of regulation, for example, it has been shown that a threshold level of gibberellins in the gynoecium is required to initiate auxin biosynthesis, providing a feedback loop (Vivian-Smith and Koltunow, 1999). Tomato fruit set can be achieved by application of auxin or gibberellin. Auxin appears to act partly through gibberellin, as it can induce gibberellin biosynthesis early during fruit development (Serrani et al., 2008), but each hormone seems to also play a specific role on its own. Auxin-induced fruit contain many more cells compared to gibberellin-induced fruits, which contain fewer larger cells (Bungerkibler and Bangerth, 1983). One of the key players in gibberellin–auxin crosstalk is an auxin response factor (ARF), *SlARF7*, which when mutated causes parthenocarpic fruit development. The mutated fruit display a thick pericarp with large cells having a similar appearance to gibberellin-induced fruit. Molecular analysis has showed that *SlARF7* was partly controlling both auxin and gibberellin signaling (de Jong et al., 2009b,^2011^). This pathway was further characterized through the analysis of the tomato *procera* parthenocarpic mutant, with a constitutive gibberellin response, and indicate that activation of the gibberellin signaling pathway after fertilization also controls *SlARF7* expression (Carrera et al., 2012).

Cytokinin levels also increase after pollination (Matsuo et al., 2012). Although cytokinins are generally considered to play a critical role in the stimulation of cell division during fruit development (Wismer et al., 1995; Srivastava and Handa, 2005), very few experimental data support the involvement of this hormone in the initial cell division phase of fruit growth (Mariotti et al., 2011). It is well known that cytokinin promotes cell proliferation at shoot apical meristems and interact closely with auxin (Murray et al., 2012) and are likely to function in a similar manner in the developing gynoecia (Lindsay et al., 2006; Bartrina et al., 2011). A recent study in *Arabidopsis* showed that cytokinin plays at least two roles during fruit development: an early proliferation-inducing role at the medial region of the developing gynoecia and a later role during formation of fruit valve margins (Marsch-Martinez et al., 2012). Finally brassinosteroids might also have a role in fruit set (Fu et al., 2008), however, the interaction with other hormones has not been investigated.

While auxin, gibberellins, and cytokinin levels are increasing at fruit set, abscisic acid (ABA) levels decrease (Hein et al., 1984; Kojima et al., 1993). Consistent with these observations, a transcriptomic analysis showed that mRNA levels of several ABA biosynthesis genes decrease after pollination, while expression of ABA degradation genes increases (Vriezen et al., 2008). ABA has also been shown to counteract the effect of gibberellin on fruit set in pea (García-Martínez and Carbonell, 1980). Expression of ethylene biosynthesis and signaling genes also decrease after pollination while in unpollinated tomato ovaries ethylene biosynthesis and signaling genes are highly expressed.

Overall, these data demonstrate that fruit set relies on a fine balance between plant hormones; the concerted action of auxin and/or gibberellin and/or cytokinin (dependency toward a specific hormone will likely depend on the plant species) will ultimately lead to activation of core cell cycle genes. We can also speculate that ABA and ethylene could have an antagonistic effect on fruit set but this will require further investigation (**Figure 1A**).

## FRUIT GROWTH

The developing seed continually sends signals to the surrounding tissue to expand and there is usually a positive correlation between seed number and fruit size (Nitsch, 1970). The developing fruit must also signals back to the rest of the plant so that it is provided with enough nutrients and does not abort. The extent of growth of the fruit from anthesis to maturity is extremely variable; in some species the fruit enlarge relatively little while in others they may increase in volume many thousand times. Unique to fleshy fruit, concomitant with cell expansion, there is an accumulation of storage products and an increase in sugar accumulation (Coombe, 1976). While fruit expansion is a key event, there is little literature covering the role of hormones in the transition for the division to the expansion phases and to the sustained growth of the fruit. Drawing on literature outside the fruit environment it is clear that cell expansion is regulated by auxin, gibberellin, and brassinosteroid (Davies, 2010; Pattison and Catala, 2012).

Cell enlargement depends on both cell wall loosening and increases in turgor pressure (Cosgrove, 2005). While auxin mostly controls cell division during fruit set, it is thought to play an important role during the growth phase by influencing cell enlargement together with gibberellins (Csukasi et al., 2011). In tomato, the maintenance of auxin gradients, through the precise localization of auxin transporters, such as the PIN transporters, will be essential for fruit growth (Pattison and Catala, 2012). A transcriptomic approach focusing on the cell expansion phase revealed that in the growing exocarp and locular tissues, a range of cell wall-related proteins are up-regulated during the expansion stage of the fruit, as well as sugar transport proteins and various glycolytic enzymes. Some genes belonging to the expansins, endo-xyloglucan transferase and pectate lyases families have been shown to be regulated by either auxin, gibberellin, or both in tomato (de Jong et al., 2011; Carrera et al., 2012). A genome-wide approach in apple, focusing on the role of auxin during cell expansion, showed that auxin action potentially involves an *ARF* gene, which is linked to quantitative trait loci (QTLs) for fruit size (Devoghalaere et al., 2012). ABA has also been associated with the expansion phase in tomato (Gillaspy et al., 1993) and ABA-deficient mutants have a reduced fruit size (Nitsch et al., 2012). The source of these hormones originates mostly from the seed and has to be transported to the surrounding tissue and/or is synthesized directly in the expanding tissue but, expect for auxin, our current knowledge is, however, limited in this area.

## FRUIT MATURATION

Fruit maturity is a developmental point where the fruit has reached the competence to ripen, but has yet to start the ripening process. Auxin and maybe cytokinin appear to be key regulators of fruit maturation. Genetic studies have shown that the tomato *ripening inhibitor* (*rin*) mutant that displays a non-ripening phenotype, have higher levels of auxin and cytokinin at breaker stage compared to wild-type fruit (Davey and Van Staden, 1978; Rolle and Chism, 1989). The suppression of a *rin*-like *MADS*-box gene in apple (Ireland et al., 2013), resulted in a maintenance of high auxin concentration during fruit maturation and fruit that did not ripen (Ireland et al., 2013; Schaffer et al., 2013). In *Arabidopsis* and *Brassica napus*, a low auxin is required for seed dehiscence (pod shatter) to occur (Chauvaux et al., 1997; Sorefan et al., 2009). A mutation in *INDEHISCENT* (*IND*) results in high levels of auxin within the valve margins of the dehiscence zone compared to wild-type controls and it has been postulated that this high intracellular auxin at least partially inhibits dehiscence (Sorefan et al., 2009). In tomato, reduction of auxin by the over-expression of a *Capsicum chinense* auxin-conjugating enzyme (*GH3*) leads to decreased auxin and an increased sensitivity to ethylene at an earlier stage of development (Liu et al., 2005). In strawberry, when achene’s are removed from immature fruit, precocious ripening of the receptacle occurs (Given et al., 1988), this ripening can be stopped by the application of exogenous auxin. During fruit growth, auxin levels in the seed are higher than in the surrounding fruit tissue (Devoghalaere et al., 2012) and this suggests as the seeds become dormant, auxin biosynthesis or transport to the rest of the fruit is inhibited, allowing the mature fruit to ripen. This appears to be supported across fruit species as addition of auxin to mature fruit invariably delays ripening (Vendrell, 1985; Manning, 1994; Davies et al., 1997; Aharoni et al., 2002). It should also be noted that although seeds have a strong influence on maturity, parthenocarpic fruit still ripen suggesting a developmental regulation may also be involved.

The role of cytokinin during fruit maturation is less well documented but cytokinin-deficient *Arabidopsis* fruit show non-synchronous ripening with fewer viable seeds compared to controls suggesting cytokinin also has a role in the regulation of silique maturation and ripening (Werner et al., 2003). Finally decreases in free cytokinin and auxin levels are also observed before ripening in orange and grape (Minana et al., 1989; Bottcher et al., 2011).

One of the challenges in future work will be to better understand the molecular mechanisms underlying fruit maturation and interaction between these hormones.

## FRUIT RIPENING/SENESCENCE

The progression of fruit ripening or senescence is a complex process involving changes to the metabolic and physiological traits of a fruit. In all fruit, in the tissue surrounding the seed, there is a color change and a change in cell wall composition causing either a dehiscence or a softening (Klee and Giovannoni, 2011). Unique to fleshy fruit there is often a breakdown of stored carbohydrates to sugars and a decrease in acidity along with an increase in flavor and aroma volatiles (Klee and Giovannoni, 2011). The control of ripening appears to be achieved predominantly through the ripening hormones ABA and ethylene (reviews: Fedoroff, 2002; Giovannoni, 2004; Setha, 2012), ethylene being the most studied. Fruit types that have a strong requirement for ethylene to ripen such as tomatoes, peaches, bananas, apples, and melon have previously been labeled climacteric and the role of ethylene in both these fruit types has been extensively reviewed (for example, Bapat et al., 2010; Paul et al., 2012). In peaches and tomato, indole-3-acetic acid (IAA) has also been reported to have some crosstalk with ethylene during ripening as (i) production of ethylene can be concomitant with an increase of IAA and (ii) auxin-signaling components can be up-regulated by ethylene and vice versa (Jones et al., 2002; Trainotti et al., 2007). In fruit that have a lower requirement of ethylene to ripen (referred as non-climacteric fruit such as grape and citrus), ABA appears to have a stronger role (Setha, 2012). It has been shown that in the climacteric fruits tomato and banana, there is an increase in ABA preceding an increase in ethylene. Exogenous application of ABA induces ethylene through the biosynthesis genes (Jiang et al., 2000; Zhang et al., 2009), while a suppression of ABA leads to a delay in fruit ripening (**Figure 1B**; Sun et al., 2012a). In the dry dehiscent fruit *Arabidopsis*, again ABA increases with silique maturation (Kanno et al., 2010) and has been linked with the promotion of dehiscence, an ethylene mediated event (Child et al., 1998; Kou et al., 2012).

While there is a considerable amount of literature on fruit ripening, researchers have often only focused on a small number of physiological changes to document the ripening process. For example color change and/or fruit firmness are often used as a surrogate for ripening, with other ripening characters completely overlooked. It is becoming clear that some ripening traits are independently controlled from each other (Johnston et al., 2009; Ireland et al., 2013). The use of single physiological marker(s) may hence lead to a misrepresentation of this complex process. Here we have summarized the literature based on how different traits respond to hormones rather than considering ripening as one single process.

### SUGAR ACCUMULATION

There is little literature on the hormonal control of starch hydrolysis and the resulting sugar accumulation. There have been a number of studies that have documented the metabolic changes that occur during maturation and ripening (Fait et al., 2008; Osorio et al., 2011,^2012^), though the link between hormonal control and metabolite accumulation is limited; however, Johnston et al. (2009) observed in apple that, while this could progress independently of ethylene, it was highly sensitive to ethylene. In melon, the application of exogenous ABA was shown to promote starch hydrolysis (Sun et al., 2012b), different from growth section, however, this was confounded by the fact that the ABA also increased the ethylene levels.

### COLOR CHANGE

Much of the literature documents the control of color change during fruit ripening. This is achieved by a combination chlorophyll loss (degreening) and production of secondary color metabolites such as carotenoids and anthocyanins. Color change in many fruit species is associated with an increase of ABA and/or ethylene. In apple, the degreening occurs independently of ethylene but ethylene can accelerate the process (Johnston et al., 2009). Citrus and melon also both require ethylene for the degreening of the skin. The production of secondary color metabolites is strongly ethylene regulated in tomato, though some intermediates can be produced in the absence of ethylene. Application of ABA to tomato fruit results in an enhanced onset of breaker stage compared to controls, further implicating ABA as being positive regulator of ripening in tomato (Buta and Spaulding, 1994). In grape and strawberry, the color change is strongly regulated by ABA (Deytieux et al., 2005; Jia et al., 2011), though application of 1-methylcyclopropene (1-MCP; an inhibitor of ethylene response) can delay this process, suggesting that ethylene may play a role (Chervin et al., 2004). There are also reports of color change being inhibited by brassinosteroids in grape and strawberry (Symons et al., 2006; Chai et al., 2013).

### CELL WALL HYDROLYSIS

There is a considerable set of literature covering ripening related changes in the cell wall (review: Brummell, 2006). Depending on the fruit type these can manifest as a formation of a dehiscence zone, or through the softening of the flesh tissue. In each case there is a suite of cell wall-related genes that are up-regulated, and in many instances each is differentially regulated. In the case of fruit softening, loss of a single gene can be compensated by other gene action (Powell et al., 2003). In apple and melon, there are both ethylene-independent and ethylene-dependent softening which can be observed in the differential regulation of cell wall-related genes. In banana, it has been shown that ABA can act synergistically with ethylene to promote softening (Lohani et al., 2004) and in grape ABA has been shown to cause fruit softening (Cantin et al., 2007).

Studies of *Arabidopsis* silique dehiscence indicate that ethylene, jasmonic acid, and ABA work in conjunction with each other to promote normal floral organ abscission *via* the up-regulation of genes like *POLYGALACTURONASE* (*ADPG1*; Ogawa et al., 2009). In *Arabidopsis*, a delayed dehiscent phenotype is associated with reduction in the ability of *Arabidopsis* fruit to produce ethylene and that a wild-type time to dehiscence can be restored with treatment of exogenous ethylene (Child et al., 1998; Patterson, 2001). Finally salicylic acid has been shown to delay softening in banana (Srivastava and Dwivedi, 2000).

### FLAVOR AND AROMA PRODUCTION

In apple, aroma volatiles are the least ethylene sensitive, and most ethylene-dependant of the ripening traits. Consistent with this, there are a significant number of publications linking the production of aroma with ethylene (Flores et al., 2002; Botondi et al., 2003; Defilippi et al., 2005; Schaffer et al., 2007). There is, however, remarkably little literature examining if other hormones contribute to the regulation of volatile production in fruit.

## SUMMARY

It is clear that there is still considerable work needed to better understand the way that hormones interact during fruit development. While there are areas that have been quite extensively covered such as fruit set and the role of ethylene in fruit ripening, there are considerable gaps in our understanding of the hormonal control and crosstalk of other areas, such as fruit expansion, endoreduplication, starch hydrolysis, and flavor development. While much of the physiology is now documented there are considerable opportunities to further our molecular understanding of these complex processes.

## Conflict of Interest Statement

The authors declare that the research was conducted in the absence of any commercial or financial relationships that could be construed as a potential conflict of interest.
